# Teachers’ Perceptions of Their Influence on Student Practices to Enhance the School Environment: A Cross-Sectional Study in Governmental General Education Schools in Al-Ahsa Province, Kingdom of Saudi Arabia

**DOI:** 10.7759/cureus.51702

**Published:** 2024-01-05

**Authors:** Yousif Elmosaad

**Affiliations:** 1 Department of Public Health, College of Applied Medical Sciences, King Faisal University, Al-Ahsa, SAU

**Keywords:** students, practices, perceptions, environment, teachers

## Abstract

Background

The school environment plays a significant role in shaping the well-being of students, as it encompasses various relationships that occur within the school community. Teachers, in particular, possess knowledge and perceptions that greatly influence their students’ behavior. This study aims to examine the perceptions of teachers regarding their impact on student practices and their efforts to enhance the school environment.

Methodology

The study employed a cross-sectional design involving general education teachers in Al-Ahsa, Saudi Arabia. Data were collected from a sample of 305 teachers through face-to-face interviews utilizing a structured questionnaire. A multistage probability sampling technique was employed to select a representative sample from the pool of school teachers. Descriptive statistics were utilized for continuous variables, while inferential statistics, such as logistic regression analyses, were employed to explore the factors influencing student practices.

Results

Overall, 264 (86.6%) school teachers had good knowledge of the school environment, and 225 (73.8%) had positive perceptions toward the school environment. The study also showed that more than two-thirds of teachers perceived that students had good practices to improve their school environment. Teachers with positive perceptions were found to be 2.84 times more likely to have positive perceptions toward students’ practices (odds ratio (OR) = 2.84, 95% confidence interval (CI) = 1.59-5.08). Teachers who had a good level of general information about the school environment were 1.6 times more likely to have positive perceptions toward students’ practices (OR = 1.63; 95% CI =1.94-2.85). Moreover, teachers who held managerial positions were 2.46 times more likely to have positive perceptions toward students’ practices (OR = 2.46; 95% CI = 1.30-4.65) when compared to those who did not hold managerial positions. The results also illustrated that high school teachers were 1.75 times more likely (OR = 1.75; 95% CI = 1.90-3.39) to have good perceptions toward students’ practices. Likewise, teachers who taught natural sciences courses had a significantly positive perception toward students’ practices to improve the school environment (p = 0.029).

Conclusions

School teachers in Saudi Arabia demonstrated a commendable level of knowledge and held a positive perception regarding school environment practices. The study findings indicate that teachers who possess a positive perception and a good level of knowledge are more inclined to harbor positive perceptions toward student practices that contribute to enhancing the school environment. Therefore, it is advisable to incorporate school environment components into the school curriculum and integrate them into teacher preparation programs.

## Introduction

The school setting encompasses various elements that impact the perceptions and interactions among teachers, students, and administrators [1]. Tapia-Fonllem et al. (2020) described the school environment as comprising factors that contribute to its own personality, spirit, and culture. They further distinguished between the school environment and the classroom environment. The classroom environment focuses on the relationships between teachers and students and among students, while the school environment encompasses a teacher’s interactions with other teachers, senior staff, and the school principal [2].

Furthermore, the school environment encompasses a broad array of dimensions and measures [3]. In a comprehensive review, Thapa et al. identified five dimensions of the school environment, namely, relationships, safety, teaching and learning, institutional environment, and school improvement process [4]. Cohen et al. (2009) explained that each of these dimensions consists of various features. Relationships pertain to the overall sense of connection within the school community. Safety includes physical aspects, such as the presence of a well-established crisis plan, as well as socioemotional elements, such as students’ attitudes, norms, and responses to bullying [5].

Furthermore, the school environment significantly impacts students. In the 21st century, there has been a growing focus on studying school environments and their connection to student well-being [6]. The school environment is recognized as an influential factor in shaping students’ eating behaviors, enabling them to make healthier food choices and lower their body mass index (BMI) [7]. Additionally, the physical activity of students is influenced by the school’s structure, such as the availability of playgrounds and access to play facilities, which play a crucial role in promoting physical activity [8-10]. School spaces are also considered didactic agents that provide optimal physical conditions for the teaching-learning process, support students’ abilities, foster their autonomy, and motivate teachers [6]. Experimental studies have identified the impact of school environments on student well-being, including positive mental health aspects and the reduction of risk behaviors [11-13].

The perceptions of teachers regarding the school environment play a crucial role in shaping students’ learning, retention, and application of knowledge, attitudes, and skills, thereby influencing their perceptions and behaviors toward their surroundings [14]. Existing literature suggests that attitudes and perceptions are significant determinants of behavior, as they are closely linked to personality and motivation [15]. Consequently, teachers’ attitudes and perceptions are frequently utilized as indicators of the school environment, as students may be unaware of many aspects of their school surroundings [2]. Recent studies have demonstrated that teachers’ positive perceptions of the school environment have numerous beneficial outcomes, including improved academic achievement, a sense of belonging to the school, and increased life satisfaction for students [16,17]. Additionally, positive perceptions are associated with reduced instances of aggression, bullying, anxiety, and depression among students [18].

Considering the aforementioned points, it can be inferred that the school environment significantly influences students’ academic achievement, well-being, and relationships with their teachers. Furthermore, teachers’ perceptions serve as a useful tool for assessing the school environment. Despite the existence of numerous studies globally examining teachers’ perceptions of the school environment, there is a lack of research from Saudi Arabia. While studies conducted in Saudi Arabian schools have focused on assessing the prevalence of obesity among students, there is a dearth of research investigating the impact of teachers’ perceptions on students’ practices for improving the school environment. Consequently, there is a need to explore the relationship between teachers’ perceptions and students’ practices. This study aims to employ quantitative methods to assess the perceptions of primary, middle, and high school teachers in Saudi Arabia regarding the school environment and investigate how these perceptions are associated with students’ practices to enhance their school surroundings.

## Materials and methods

Study design

This study employed a cross-sectional approach to investigate how school teachers’ perceptions are associated with students’ practices to enhance their school surroundings. Ethical approval was obtained from the Ethics Committee at King Faisal University-Deanship of Scientific Research (approval number: 1896/2023). Before their participation, all participants signed an informed consent form in either Arabic or English. Throughout the study, ethical considerations and confidentiality of participants’ information were strictly maintained through the use of an anonymized data collection tool comprising various variables to assess both teachers’ perceptions and students’ practices.

Study participants

Governmental primary, middle, and high schools in Al-Ahsa province were surveyed. Male and female (305) teachers were surveyed at the time of the study.

Sample techniques

The study population comprised primary, middle, and high school teachers who were working in Al-Ahsa province. According to the General Directorate of Education in Al-Ahsa Governorate, the total number of teachers is 17,148, of whom 8,035 (46.9%) are primary school teachers, 4,812 (28.1%) are middle school teachers, and 4,301 (25.0%) are high school teachers. A multistage probability sampling technique was used for selecting a representative sample from the study population. The sample size was determined appropriate to yield significant results with a 95% confidence level and a 5% margin of error. All primary, middle, and high schools for both genders in the province were included in the study. A sample size of 364 teachers was obtained using the Leslie Kish formula: n = Z^2^PQ/D^2^, where Z is the corresponding value to 95 % confidence limits (1.96), P is teachers’ perceptions (0.74) [19], Q is I − P (0.16), D is the desired margin of error (absolute precision) (0.05), and effect size of 2.

A total of 364 teachers were included in the study, distributed proportionally across different school levels. Specifically, 171 (46.9%) teachers were selected from primary schools, 102 (28.1%) from middle schools, and 91 (25.0%) from high schools in the Al-Ahsa province. Schools participating in the study were proportionally selected across different school levels and were chosen randomly from each category. All teachers in the selected schools were considered potential study participants unless they expressed their unwillingness to participate. The overall response rate was 83.8%, with a higher response rate among middle school teachers (98.0%), followed by high school teachers (86.8%), and a lower response rate among primary school teachers (73.7%).

Data collection instruments

After deciding on what type of data to collect, the instrument was constructed and validated to collect quantitative data. It consisted of four sections. The first section contained items referring to teachers’ demographic characteristics, including gender, grade, age, marital status, specialty, average monthly income, place of residence, teaching experiences, and managerial position. The second section consisted of five (yes or no) questions assessing teachers’ general information on the school environment. The question answered with “yes” was given one point, and zero points if the answer was “no.” The responses were dichotomized by assuming the mean was a cutoff point.

The third section consisted of eight items based on a five-point Likert scale ranging from one (strongly disagree) to 5 (strongly agree) for teachers’ perceptions toward the environment. These items referred to the school facilities, students’ support, a feeling of acceptance and respect, school social environment, attention to the student’s behavior, facilitating academic success for students, cultural acceptance of student’s differences, and feeling of an impact in shaping the school environment. A composite score was generated. The scores were dichotomized by using the mean as a cutoff point [20].

The fourth section referred to perceptions of students’ practices to improve the school environment. It consisted of seven yes/no questions referring to participation in environmental activities, group learning activities, adherence to policies and guidelines at school, the collaboration of students with their schoolmates and school staff to shape a healthy and supportive school environment, students’ involvement in decision-making, organizing health education and promotion campaigns in schools, and being pleasant and friendly to their peers and teachers. The questions answered with “yes” were given one point, and zero points if the answer was “no.” The scores were dichotomized by assuming the mean as a cutoff point. The last section asked for the sources of information used by teachers to improve their knowledge related to the school environment. The instrument was piloted among school teachers for validation. The obtained Cronbach’s alpha was 0.72. The instrument or questionnaire was initially developed in English and subsequently translated into Arabic to ensure better comprehension among participants.

Data collection and analysis

The data obtained from the study participants, following their consent, was collected through face-to-face interviews using the study instrument or questionnaire items. The collected data were then analyzed using SPSS version 24 (IBM Corp., Armonk, NY, USA). Two different individuals entered the data to ensure accuracy.

Descriptive statistics were employed to calculate the mean (standard deviation) for continuous variables, whereas proportions were used to analyze categorical variables. This analysis included an assessment of teachers’ perceptions and general information regarding the school environment.

Inferential statistics, specifically logistic regression analyses, were conducted to explore the predictors of students’ practices in improving the school environment. The odds ratio (OR) with a 95% confidence interval (CI) and corresponding p-value of less than 0.05 were considered statistically significant in this context.

## Results

A total of 305 participants completed the survey, with 196 (64.3%) males and 109 (35.7%) females. Within the sample, 38 (12.5%) teachers were classified as young, falling within the age range of 26-33 years. The next largest age group was 34-41 years, accounting for 84 (27.5%) participants, while 42 years and above constituted 183 (60.0%) participants.

Among the 305 participants, 251 (82.3%) were married. Regarding subject specialization, 182 (62.3%) participants were natural sciences teachers, followed by 42 (13.8%) who were linguistics teachers.

Concerning monthly income, 202 (66.2%) teachers earned more than 11,001 SR per month. Additionally, 207 (67.9%) study participants worked and resided in urban areas. Regarding teaching experience, 229 (75.1%) participants had been teaching for 11 years or more, while only 75 (24.6%) held an administrative position (Table 1).

**Table 1:** Sociodemographic characteristics of the study participants (n = 305). The mean age of the respondents was 42.6 ± 7.3 years, with 44.2 (±7.2) males and 39.8 (±6.7) females.

Category	Response options	N	%
Age group	26–33 years	38	12.5
	34–41 years	84	27.5
	42–49 years	128	42
	>50 years	55	18
Gender	Male	196	64.3
	Female	109	35.7
Marital status	Single	54	17.7
	Married	251	82.3
Specialty	Natural Sciences	190	62.3
	Linguistics	42	13.8
	Business and Computer Science	11	3.6
	Social Sciences	18	5.9
	Islamic Studies	11	3.6
	Education & Teaching	33	10.8
School level	Primary school	126	41.3
	Middle school	100	32.8
	High school	79	25.9
Average monthly income	4,000–6, 000 SR	8	2.6
	6,001–9, 000 SR	42	13.8
	9,001–11, 000 SR	53	17.4
	More than 11,001 SR	202	66.2
Place of residence	Village	98	32.1
	City	207	67.9
Years of teaching experience	Under 5 years	33	10.8
	5–10 years	43	14.1
	11–15 years	76	24.9
	16–20 years	78	25.6
	21 years and more	75	24.6
Managerial position	Yes	230	75.4
	No	75	24.6

Table 2 illustrates the responses to the items related to the teacher’s knowledge of the school environment. The majority 284 (93.1%, 95% CI = 90.5-95.7) of the study participants knew that the school environment refers to the relationships between teachers, students, and school facilities, and 274 (89.8%, 95% CI = 86.6-93.0) knew that school policies and guidelines support students to reach the expected standard of behavior on the school environment. Around 268 (87.9%, 95% CI = 84.5-91.3) participants recognized that schools offer an additional useful platform to deliver health education messages in the school environment.

**Table 2:** Teachers’ knowledge of school environment (n = 305). The mean teacher knowledge score of the school environment was 4.33 ± 1.54, with scores ranging from 1 to 5.

Variables	Count	%	95% CI
The school environment refers to the set of relationships that occur among teachers, students, and school facilities (Yes)	284	93.1	90.5–95.7
The school offers an additional useful platform to deliver health education messages in the school environment (Yes)	268	87.9	84.5–91.3
School policies and guidelines support students to reach the expected standard of behavior in the school environment (Yes)	274	89.8	86.6–93.0
Reduction of violent behaviors in schools is due to changes in the physical environments (Yes)	249	81.6	77.6–85.6
Social interactions in the school environment influence the education objectives (Yes)	245	80.3	76.1–84.5
Total average	264	86.6	83.0–90.2

Regarding reducing violent behaviors in schools, 249 (81.6%, 95% CI = 77.6-85.6) study participants understood that changes in the physical environments play a key role in reducing violent behaviors among school students. Moreover, 245 (80.3%, 95% CI = 76.1-84.5) knew that social interactions in the school environment influence educational objectives.

Table 3 discloses the teachers’ perceptions of the school environment. Accordingly, the mean score was 2.91, which was greater than the expected average mean value of 2.50. The results showed that 269 (88.2%) study participants perceived that the school teachers were paying too much attention to the students’ behavior. Moreover, around 250 (82.0%) study participants perceived that the school teachers help the students when they have personal and social problems at school. Similarly, the teachers felt accepted, respected, included, and supported in the school.

**Table 3:** Teachers’ perceptions of the school environment. The overall score of the participant’s perception was calculated by adding the score for each question, and the maximum score for the perception variable was 8. The response to the questions was on a five-point Likert scale ranging from 1 (strongly disagree) to 5 (strongly agree).

Teachers’ perception	Agree	Mean	SD
	N (%)	Out of 4	
School facilities are adequate for implementing a variety of student activities	200 (65.6)	2.85	1.2
I feel teachers help students when they have a personal and social problem at school	250 (82.0)	3.16	0.9
I feel accepted, respected, included, and supported in the school	242 (79.3)	3.16	0.9
The social environment of the school reflects the quality of relationships between members of the school community	173 (56.7)	2.08	1.2
Teachers pay too much attention to students’ behavior	269 (88.2)	3.33	0.8
I feel teachers pay enough attention to support students to succeed academically	199 (65.2)	2.64	1.4
I think that most teachers accept cultural and student differences	235 (77.0)	3.02	1
I believe that teachers play a significant role in shaping the school environment	233 (76.7)	3.02	0.9
Total average	225 (73.8)	2.91	1.05

Regarding cultural and student differences, approximately 235 (77.0%) study participants perceived that most teachers accept and embrace these differences. They believed that such acceptance has a significant impact on shaping the overall school environment. However, only 173 (56.7%) participants perceived that the social environment within the school accurately reflects the quality of relationships among the members of the school community.

The results revealed that 219 (71.9%) teachers perceived that students had good practices that improved their school environment. Moreover, 219 (71.9%) teachers perceived that students had good practices that improved their school environment. Overall, 279 (91.5%, 95% CI = 88.6-94.4) teachers perceived students participating in group learning activities at school, and 267 (87.5%, 95% CI = 84.0-91.0) teachers perceived that students collaborate with their colleagues and school staff to shape a healthy and supportive school environment. Moreover, 266 (87.2%, 95% CI = 83.7-90.7) teachers perceived students as pleasant and friendly to their peers and teachers, and 145 (47.5%, 95% CI = 42.3-52.7) teachers perceived that students were involved in organizing health education and promotion campaigns in the school. While only 101 (33.1%, 95% CI = 28.2-38.0) teachers perceived that students engaged in decision-making in schools (Table 4).

**Table 4:** The descriptive statistics of teachers’ perceptions regarding students’ practices to enhance the school environment. Teachers’ perceptions of students’ practices to improve the school environment. It consists of eight Yes/No questions. The response to the questions was in the form of Yes or No and one point was allocated for each correct answer.

Teachers’ perceptions toward students’ practices	Count	%	95% CI
Participate in activities that improve the school environment	248	81.3	77.2–85.4
Participate in group learning activities at school	279	91.5	88.6–94.4
Adhere to policies and guidelines that govern the expected standard of behavior at the school	231	75.7	71.2–80.2
Collaborate with schoolmates and school staff to shape a healthy and supportive school environment	267	87.5	84.0–91.0
Involved in decision-making in the schools	101	33.1	28.2–38.0
Pleasant and friendly to their peer and teachers	266	87.2	83.7–90.7
Involved in organizing health education and promotion campaigns in the school	145	47.5	42.3–52.7
Total average	219	71.9	69.2–76.6

**Figure 1:**
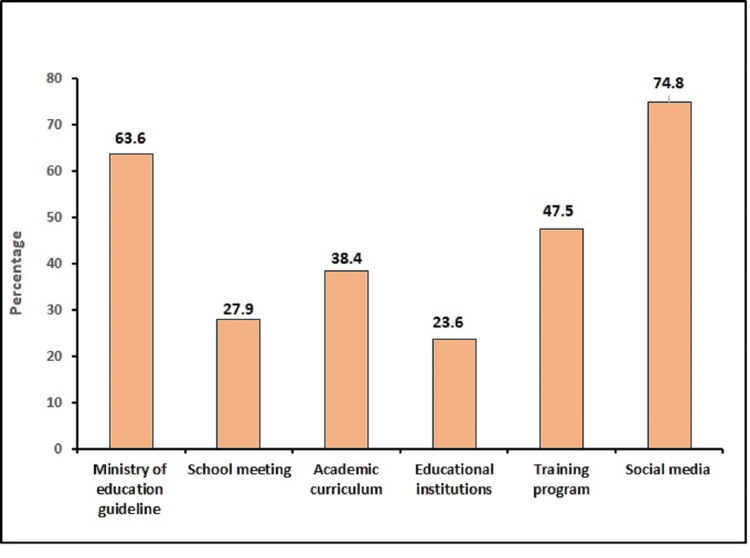
The sources of information used by teachers to improve their knowledge related to the school environment.

Figure 1 shows the sources of information used by teachers to improve their general knowledge related to the school environment. The main sources of information about the school environment included social media, Ministry of Education guidelines related to the school environment, training programs, academic curricula, school meetings, and educational institutions. The most important source of information was social media at 228 (74.8%), followed by Ministry of Education guidelines related to the school environment at 197 (63.6%), and training programs at 145 (47.5%).

Multiple logistic regression was performed to determine the association between teachers’ perceptions and students’ practices for improving the school environment adjusted by sociodemographic variables. The results indicated a significant association between teachers’ perceptions and students’ practices to improve the school environment. Specifically, teachers with positive perceptions were found to be 2.84 times more likely to have positive perceptions toward students’ practices that can improve the school environment (OR = 2.84, 95% CI = 1.59-5.08). Teachers who had a satisfactory level of general information about the school environment were 1.6 times more likely to have positive perceptions toward students’ practices that can improve the school environment (OR = 1.63; 95% CI = 1.94-2.85). Moreover, teachers who held managerial positions were 2.46 times more likely to have positive perceptions toward students’ practices (OR = 2.46; 95% CI = 1.30-4.65) compared to those who did not hold managerial positions. Our results also illustrated that high school teachers were 1.75 times more likely to have a good perception of students’ practices (OR = 1.75; 95% CI = 1.90-3.39); likewise, teachers who taught natural sciences courses significantly had positive perceptions toward students’ practices that can improve the school environment (p = 0.029). In addition, older teachers and teachers living in cities significantly had higher odds of positive perceptions toward students’ practices. Gender, marital status, and teaching experiences were not significant predictors of students’ practices (Table 5).

**Table 5:** Association between teachers’ perceptions and students’ practices for improving the school environment adjusted by knowledge and sociodemographic variables. *: Statistically significant at p < 0.05. Independent variables: teacher perceptions. Dependent variables: teacher perception of students’ practices. Ref.: reference category.

Variable	Responses	β	COR (95% CI)	β	AOR (95% CI)
Students’ practices	Poor	Ref.			
	Good	1.33	3.77 (2.33–6.09)*	1.05	2.84 (1.59–5.08)*
Teacher background information	Poor			-	
	Fair	0.58	1.79 (1.13–2.84)*	0.49	1.63 (1.94–2.85)*
Age group	26–33 years	Ref.		-	
	34–41 years	0.13	1.14 (0.52–2.46)	0.48	1.62 (0.51–5.19)
	42–49 years	-0.23	0.80 (0.38–1.67)	0.32	1.37 (0.39–4.83)
	>50 years	0.36	1.43 (0.62–3.28)	1.23	3.45 (3.80–4.52)*
Gender	Male		Ref.		
	Female	0.11	1.12 (0.70–1.79)	0.04	1.04 (0.53–2.03)
Marital status	Single	Ref.			
	Divorced	-0.35	0.71 (0.12–4.04)	-1.03	0.36 (0.05–2.82)
	Married	0.32	1.38 (0.72–2.62)	0.55	1.74 (0.72–4.22)
Specialty	Natural Sciences	Ref.			
	Linguistics	-1.10	0.33 (0.17–0.65)*	-0.83	0.44 (0.21–0.94)*
	Business and Computer Science	-2.77	0.06 (0.01–0.49)*	-2.8	0.06 (0.01–0.65)
	Social Sciences	-0.49	0.61 (0.25–1.54)	-0.91	0.40 (0.14–1.16)
	Islamic Studies	-0.89	0.41 (0.13–1.25)	-0.25	0.78 (0.22–2.74)
	Education & Teaching	-1.27	0.28 (0.13–0.60)*	-0.91	0.41 (0.17–0.95)*
School level	Primary school	Ref.			
	Middle school	0.12	1.13 (0.60–2.09)	-0.16	0.85 (0.38–1.94)
	High school	0.74	2.10 (1.22–3.61)*	0.56	1.75 (1.90–3.39)*
Place of residence	Village	Ref.			
	City	0.53	1.70 (1.03–2.81)*	0.773	2.17 (1.17–4.02)*
Years of teaching experience	Under 5 years	Ref.			
	5–10 years	0.35	1.42 (0.57–3.54)	0.50	1.65 (0.51–5.32)
	11–15 years	0.09	1.09 (0.48–2.51)	0.05	1.05 (0.29–3.74)
	16–20 years	-0.33	0.72 (0.31–1.65)	-0.67	0.51 (0.1–32.07)
	21 years and more	0.01	1.01 (0.44–2.31)	-0.85	0.43 (0.10–1.81)
Managerial position	No	Ref.			
	Yes	0.81	2.25 (1.33–3.82)*	0.90	2.46 (1.30–4.65)*

Teachers who had positive perceptions, held managerial positions, taught at the high school level, had a satisfactory level of general information about the school environment, were older, and lived in cities had a significant impact on students’ practices related to the school environment. Surprisingly, gender and teaching experiences were not significant predictors of students’ practices (Table 5).

## Discussion

The school environment is a principal factor when evaluating students’ well-being. It refers to the sum of relationships between the school community members that are determined by the personal, structural, and functional factors of the school. Teachers’ perceptions are generally dependent on competencies, self-efficacy, experience, and continuous development as determinants of students’ behavior [14,21]. Several studies have been undertaken across various regions worldwide to examine the association between teachers’ perceptions of the school environment and their teaching practices. This study sought to examine the impact of teachers’ perceptions of the school environment on students’ practices aimed at improving the school environment. In this study, most participants possessed good knowledge about the various aspects of the school environment, such as student well-being, relationships, policies and guidelines, physical environments, social interactions, and effective platforms for delivering health education messages. Comparable results have been reported in previous studies conducted in India [22], Malaysia [23], and Saudi Arabia [24]. This could be attributed to the comprehensive curriculum in teacher training institutions and schools, which provides ample information on the school environment. Additionally, professional development programs and media exposure contribute to enhancing teachers’ performance in all educational aspects.

Regarding teachers’ perceptions, the study revealed that teachers hold positive views of the school environment. They believe that schools play a supportive role in students’ personal and social issues and that they feel accepted, respected, and inclusive of cultural and individual differences. Furthermore, teachers perceive their influence on shaping the school environment positively. These findings align with a study conducted in Bangladesh, which reported positive perceptions among teachers regarding conducive school environments [25]. Similarly, Susanoo et al. (2018) reported that teachers have positive perceptions regarding certain components of the school environment, such as valuing students’ progress and providing guidance to students facing difficulties. Moreover, the results indicate that teachers’ knowledge positively influences their perceptions, consistent with the findings of Bahmaid et al. (2018), who suggested that increased knowledge promotes positive perceptions [26].

In this study, more than two-thirds of teachers perceived that students exhibit good practices in improving the school environment. These practices include engaging in group learning activities, collaborating with peers and school staff, maintaining a healthy and supportive school environment, and displaying friendly behavior toward peers and teachers. These results align with the theoretical foundation of the theory of planned behavior, which posits that perceptions ultimately influence intended and actual behaviors [27]. However, only one-third of teachers perceived that students engage in decision-making processes in schools, indicating a need to improve leadership styles in schools. Involving students in decision-making processes, particularly in matters that directly concern them, can significantly impact behavior, performance, and overall school management.

A multiple logistic regression model, adjusted for sociodemographic variables and level of knowledge, was employed to determine the association between teachers’ perceptions and students’ practices. The results indicated a significant association between positive teacher perceptions, good knowledge, and positive perceptions of student practices for improving the school environment. This suggests that teachers’ knowledge and positive perceptions influence students’ practices. These findings are consistent with previous studies demonstrating that teacher quality and perceptions have a significant impact on student learning and academic performance [28]. Furthermore, the model revealed that teachers in managerial positions, those teaching natural sciences, and those residing in urban areas are more likely to hold positive perceptions of student practices. While there is some controversy regarding the influence of teacher characteristics on student practices, some studies emphasize their importance in student learning outcomes [29]. These characteristics have a significant impact on students’ knowledge, perceptions, and practices related to the school environment.

Limitations and strengths

The present study has several limitations that should be considered. First, the cross-sectional design only enables a picture of relationships at a certain period in time and does not allow any conclusions about causal effects. Furthermore, the relationship between teacher perceptions and students’ practices would probably be reciprocal. Additionally, this study was limited only to teachers working in government schools.

On the other hand, our study investigated the relationship between teachers’ perceptions of how they influence students’ practices for improving the school environment, assessed the teachers’ level of general information and perceptions, and determined the associations between teachers’ perceptions and students’ practices. This study provides useful data for guiding the initiation of an effective improvement policy to integrate school environment components into the curriculum and teacher preparation programs. Moreover, it lays the groundwork for future research at a national level to develop a holistic and deep understanding of the role of teachers’ perceptions regarding both the government and private school teachers on the topic of school’s physical and psychosocial environment.

Moreover, the study underpinning the importance of the school leadership approach in impacting various aspects of the school environment from academic achievements to the overall school culture and students’ practices for improving their school environment ensures community engagement and creates inclusive environments.

## Conclusions

This study sheds light on the association between teachers’ perceptions of the school environment and their students’ practices for improving the school environment. The findings highlight the importance of teachers possessing good knowledge and positive perceptions of the school environment. Moreover, the study reveals that teachers in managerial positions, those teaching natural sciences, and those residing in urban areas are more likely to hold positive perceptions of student practices. These findings emphasize the need to integrate components of the school environment into curricula and teacher preparation programs.

Finally, in this century, schools requiring innovative leadership approaches need to set visions, adapt to contemporary challenges, embrace technology as an integral part of education, guide school staff, involve students and community through strategic planning, as well as need to focus on the well-being of staff and students by adopting strategies to promote mental, emotional, and physical health within the school environment.
